# Postoperative Patient-Reported Pain and Opioid Consumption After Total Hip Arthroplasty: A Propensity Score-Matched Comparison of the Direct Superior and Posterior Approaches

**DOI:** 10.3390/jcm14051400

**Published:** 2025-02-20

**Authors:** Seok Ha Hong, Kang Hun Yu, Seung Beom Han

**Affiliations:** Korea University Anam Hospital, Seoul 02841, Republic of Korea

**Keywords:** direct superior approach, opioid consumption, patient-reported pain, total hip arthroplasty

## Abstract

**Background:** The direct superior approach (DSA), a muscle-sparing technique derived from the posterior approach (PA), has received little attention despite its potential advantages. This study compared the impact of the DSA and PA on patient-reported pain and postoperative opioid consumption with medical and surgical complications. **Methods:** We reviewed 451 primary total hip arthroplasties for osteonecrosis of the femoral head and osteoarthritis between January 2016 and December 2022, which were categorized as DSA or PA. Demographic data, including age, sex, preoperative opioid usage, smoking status, chronic alcoholism, and underlying diseases, were collected. Propensity score matching balanced the DSA and PA groups. The maximum and minimum pain score on the postoperative day (POD) and total opioid consumption were compared between the two groups. Inflammation-related serum markers, medical and surgical complications, and the length of hospital stay were also analyzed. **Results:** After matching, 139 patients were included in each group. Patients with the DSA reported a lower average maximum pain on POD #1 and #4 and a lower average minimum pain on POD #1, #2, and #4. The DSA group showed a significant reduction in opioid consumption. In addition, the DSA led to a significant reduction in C-reactive protein (CRP) on POD #5, 14, and 28 after the index surgery compared to the PA. Postoperative delirium (3.6 vs. 9.4%, *p* = 0.05) and length of stay (5.4 vs. 8.4 days, *p* < 0.001) were significantly different between the two groups. No significant differences were observed in chronic opioid use, medical complications, or other surgical complications. **Conclusions:** The DSA was associated with lower patient-reported pain and a marked reduction in opioid consumption, delirium, and length of hospital stay.

## 1. Introduction

The landscape of total hip arthroplasty (THA) has continually evolved, driven by the pursuit of swifter recovery and enhanced pain management, as highlighted by Penenberg et al. [1]. Postoperative pain control is a key factor influencing patient outcomes, and various strategies have been explored to optimize the recovery process [2]. These include advancements in anesthesia techniques, perioperative pain management protocols, and surgical approaches [3]. Regional anesthesia, such as spinal or epidural anesthesia, has been associated with reduced opioid consumption and improved early recovery compared to general anesthesia [4].

In addition, the choice of surgical approach plays a crucial role in postoperative pain control and recovery. While an ideal approach has yet to be established, minimally invasive surgical techniques in THA (MIS-THA) have gained popularity due to their potential benefits in terms of reducing soft tissue trauma, lowering opioid requirements, and promoting faster rehabilitation [5].

Despite extensive investigations into MIS-THA, there is no consensus on a singular preferred approach for THA. While recent studies have shown that the direct anterior approach (DAA) MIS-THA technique reduced postoperative pain and opioid consumption, the direct superior approach (DSA) has yet to receive the same level of attention [6]. The DSA is a muscle-sparing hip approach, derived from the posterior approach (PA), designed to preserve the iliotibial band and minimize damage to short external rotators [7,8]. Notably, the DSA is a modification of the PA, offering a shallower learning curve for those familiar with the PA technique [9,10]. Furthermore, it provides flexibility, allowing for extending the incision if necessary.

Therefore, this study aimed to compare the direct superior approach (DSA) and the posterior approach (PA) in terms of patient-reported pain, postoperative opioid consumption, and length of hospital stay. Using propensity score matching (PSM) to minimize confounding, we hypothesized that the DSA would result in lower pain scores, reduced opioid use, and faster functional recovery compared to the PA.

## 2. Method

### 2.1. Patient Demographics

This study was conducted in accordance with the guidelines of the Declaration of Helsinki and approved by the Institutional Review Board of Korea University Anam Hospital (protocol code: 2023AN0471, date of approval: 26 October 2023). This study reviewed all patients who underwent a primary THA for the diagnosis of osteonecrosis of the femoral head and primary or secondary osteoarthritis. The study period spanned January 2016 to December 2022. Patients who had undergone previous hip surgery, those with severe hip dysplasia requiring femur osteotomy or adductor tenotomy, those with pathologic fractures, and those with femur neck fractures were excluded from the study.

Patient demographics, including age, sex, preoperative opioid usage, smoking status, chronic alcoholism, underlying diseases (hypertension, diabetes mellitus (DM), Parkinson’s disease, dementia, angina, and stroke), body mass index (BMI), hip pathology, history of previous lumbar spine fusion, and American Society of Anesthesiologists (ASA) score were included in this study. The type of anesthesia used was classified as either regional or general. Additionally, head sizes ranging from 28 mm to 36 mm were assessed, and the use of the dual mobility construct was analyzed.

### 2.2. Cohort Matching

Propensity score matching (PSM) was employed to account for baseline demographic differences between patients in the DSA and PA groups. PSM considered factors such as sex, age, smoking status, chronic alcoholism, preoperative opioid use, BMI, underlying diseases, diagnosis, history of previous lumbar spine fusion, head size, dual mobility construct, and ASA score. A 1:1 matching of the DSA and PA was performed using a nearest-neighbor matching algorithm, with a maximum caliper of 0.05 for the hazard components.

### 2.3. Surgical Approaches

Until 2019, the senior author primarily employed the PA for THA procedures. In 2019, the senior author transitioned to the direct superior approach (DSA) for all primary THA procedures, replacing PA as their primary approach. No specific exclusion criteria were applied to the DSA group. The indications for both approaches were consistent, with the DSA used in all primary THA cases starting from 2019.

### 2.4. Direct Superior Approach

An 8 cm incision was made starting from the middle and posterior thirds of the greater trochanter, 45° backward and upward. After the fascia of the gluteus maximus was incised without extending to the iliotibial band, the gluteus maximus muscle fibers were divided longitudinally, exposing the pericapsular fat along the posterior border of the greater trochanter. The piriformis and conjoined tendons were detached from their insertions into the posterior greater trochanter. The quadratus femoris remained intact. After the posterior hip capsule was completely exposed, the superior capsulotomy capsule was incised anteriorly and posteriorly. After component implantation, a direct side-to-side repair of the capsule was performed, and the obturator internus and piriformis tendons were attached to the posterior aspect of the gluteus medius tendon.

### 2.5. Pain Management Protocol

All patients received periarticular injections of ropivacaine (150 mg) and morphine (7.5 mg) during surgery. Postoperatively, a standardized pain management protocol was followed, consisting of the scheduled administration of acetaminophen (325 mg) combined with tramadol (37.5 mg) three times a day and celecoxib (200 mg) once daily. For moderate-to-severe pain, additional oral opioid medications or intramuscular opioid injections were administered as needed.

### 2.6. Patient-Reported Pain and Opioid Consumption

Each participant in the study underwent multiple daily pain assessments during the postoperative days (PODs) following the index surgery, in accordance with the hospital’s standard nursing protocol. Pain levels were self-reported by patients on a scale of 0 to 10, where 0 denoted no pain and 10 represented the maximum pain level. The recorded data included the highest and lowest reported pain levels on each POD for each patient. Additionally, the study comprehensively quantified inpatient opioid consumption, including both oral medications and intramuscular injections, which was measured in terms of oral morphine equivalents (OME) [11]. The study also investigated whether patients received opioid prescription refills after 90 days and between six months and 1 year after the index surgery.

### 2.7. Postoperative Outcomes

This study included patients with multiple postoperative outcomes. First, serum C-reactive protein (CRP) and erythrocyte sedimentation rate (ESR) levels were measured at POD 2,5, 14, and 28 days to assess the invasiveness and infection-related complications of the surgical procedure. In addition, major medical complications that occurred within 6 months post-surgery were recorded, including cardiovascular events (acute myocardial infarction), respiratory issues (pneumonia), genitourinary complications (acute renal failure and urinary tract infection), and neurological complications (cerebrovascular infarction and delirium). Surgical complications, including intraoperative fractures, periprosthetic joint infections, periprosthetic fractures, early dislocations, the need for revision surgery in cases of implant malposition leading to dislocation, periprosthetic joint infection, and periprosthetic fracture, were documented. The Harris Hip Score (HHS) and Western Ontario and McMaster Universities Osteoarthritis score (WOMAC) 6 months and 1 year post-surgery, the length of hospital stay, and 90-day readmission rates were also analyzed.

Discharge was considered when patients were able to walk a certain distance (e.g., 10 m) with the assistance of aids (e.g., crutches or a walker), their pain levels were low enough to not require opioid injections, and no other medical complications had occurred. The rehabilitation protocol involved walking exercises and abductor muscle strengthening. Follow-up visits were scheduled at 2 weeks, 6 weeks, 3 months, 6 months, and 1 year post-surgery.

### 2.8. Statistical Analysis

To compare the baseline characteristics and clinical outcomes between patients who underwent the DSA and those who underwent the PA, we employed appropriate statistical tests. Categorical variables were assessed using the Pearson’s chi-square or Fisher’s exact tests, whereas continuous variables were analyzed using independent *t*-tests. PSM was used to match the DSA and PA groups to minimize potential confounding variables. All statistical analyses were performed using the R software package v.3.3.3, with statistical significance set at *p* < 0.05.

## 3. Results

### 3.1. Patient Selection

**Figure 1:**
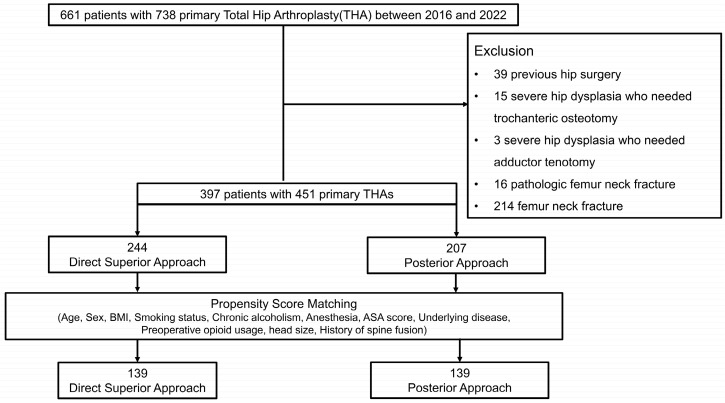
Patient selection flowchart.

Of the initial 738 primary THAs, 451 met the rigorous inclusion and exclusion criteria, as depicted in the flowchart below (Figure 1). Table 1 shows the baseline patient demographics and that there were significant differences in anesthesia, ASA score, DM, and femoral head size between the two approaches. To address these variations, PSM was employed, resulting in the selection of 139 hips from each group. After matching, the patients’ characteristics, including age, sex, smoking status, chronic alcoholism, preoperative opioid usage, anesthesia, diagnosis, and underlying diseases, were effectively balanced, with standardized mean differences of less than 0.15 (Table 2).

### 3.2. Patient-Reported Pain and Inpatient Opioid Consumption

**Figure 2:**
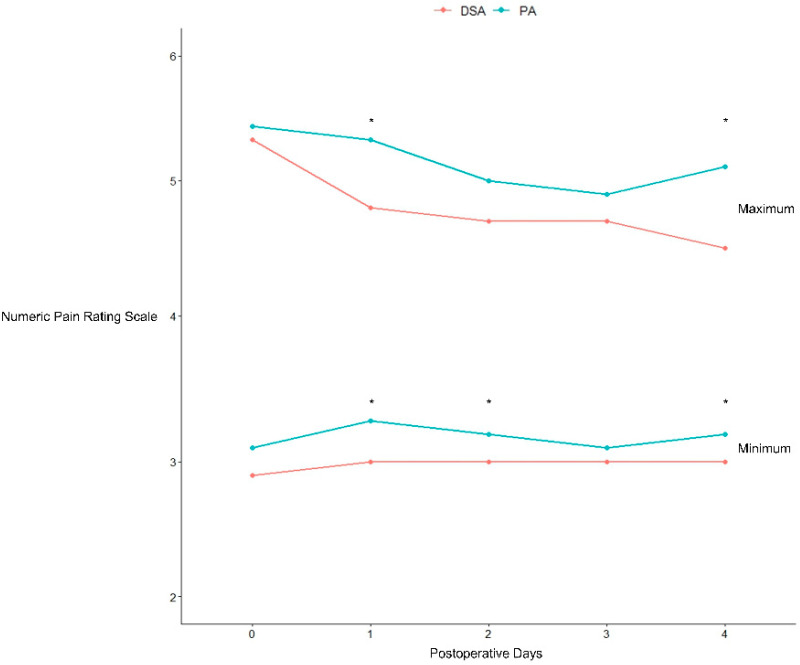
The upper half of the graph represents the maximum NRS scores reported by patients each postoperative day, while the lower half of the graph represents the minimum NRS scores. The red line indicates the direct superior approach (DSA), and the aqua line indicates the posterior approach (PA). Asterisks (*) denote time points where the difference between the two groups was statistically significant (*p* < 0.05).

Patients that underwent the DSA reported a lower average maximum pain on POD #1 (4.8 ± 1.1 vs. 5.3 ± 1.5, *p* < 0.01) and POD #4 (4.5 ± 0.8 vs. 5.1 ± 1.4, *p* < 0.01) and a lower average minimum pain on POD #1 (3.0 ± 0.6 vs. 3.3 ± 0.9, *p* = 0.04), POD #2 (3.0 ± 0.5 vs. 3.2 ± 0.8, *p* = 0.02), and POD #4 (3.0 ± 0.6 vs. 3.2 ± 0.7, *p* = 0.01) (Table 3) (Figure 2). Moreover, a significant difference in OME was observed between the two groups (108 vs. 194, *p* = 0.002). In contrast, there were no differences in outpatient opioid consumption after 90 days or between 90 and 180 days after the index surgery (2 vs. 1 and 1 vs. 0, respectively, *p* > 0.9).

### 3.3. Postoperative Outcomes

**Figure 3:**
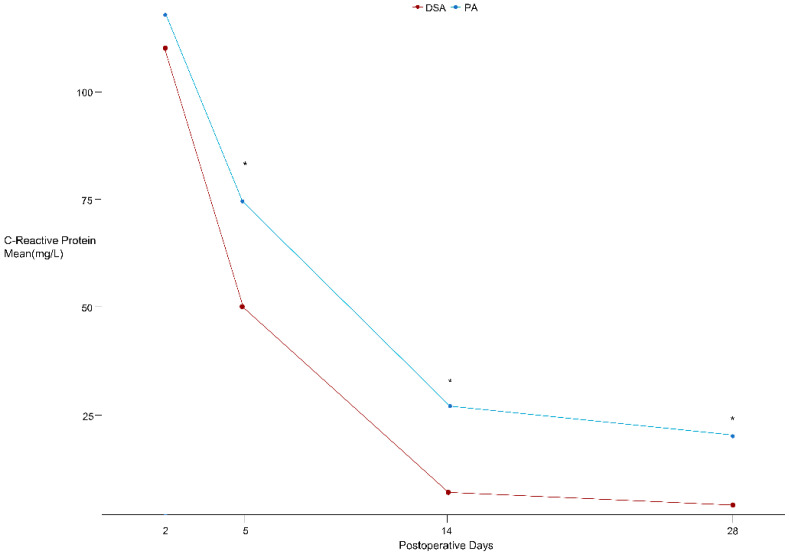
This graph compares CRP levels on postoperative days 2, 5, 14, and 28. The red line represents the direct superior approach (DSA), while the aqua line represents the posterior approach (PA). Asterisks (*) indicate time points where the difference between the two groups was statistically significant (*p* < 0.05).

CRP levels was significantly decreased in the DSA group on PODs 5, 14, and 28 compared to those in the PA group (Figure 3 and Table 4). No major medical complications, including myocardial infarction, pneumonia, acute kidney injury, urinary tract infection, cerebrovascular infarction, or mortality, were recorded in either group (Table 5). Remarkably, postoperative delirium was lower in the DSA group (3.6 vs. 9.4%, *p* = 0.05). Regarding surgical complications, one revision for a postoperative periprosthetic fracture was performed in each group, and although there was a difference in early dislocation rates, it did not reach statistical significance (2.2 vs. 5.6%, *p* = 0.15). Also, no significant differences were observed in the HHS and WOMAC scores between the two groups at both the 6-month and 1-year post-surgery intervals. Notably, a substantial reduction in the length of stay was observed in the DSA group compared with the PA group (5.4 vs. 8.4 days, *p* < 0.001).

## 4. Discussion

In the current study, we conducted an evaluation of the postoperative outcomes for two matched groups of patients who underwent primary THA using either the DSA or PA. Our findings revealed that the DSA group exhibited lower levels of patient-reported pain and a significant reduction in their postoperative length of stay and CRP levels. Additionally, inpatient postoperative opioid consumption and delirium rates were lower in the DSA group. However, there was no statistically significant difference in outpatient opioid refill rates at 90 days or between 6 months and 1 year after the index surgery, nor was there a difference in long-term functional outcomes.

The DSA is a tissue-sparing technique that preserves the iliotibial band and quadratus femoris through the use of a smaller incision [7,8,12]. Our hypothesis is that the perceived reduction in pain and faster recovery associated with the DSA is mainly attributable to the preservation of the iliotibial band (IT band) and the utilization of a modified capsular repair method [13]. In a retrospective comparative study, over 3000 patients who underwent bilateral total hip arthroplasty (THA) through the DSA, with and without IT band preservation, were surveyed about their pain perception. The results indicated that the majority of patients preferred the surgery with IT band preservation [14]. Subsequently, IT band and soft tissue preservation in the DSA has led to a dramatic decrease in postoperative NRS pain scores (maximum and minimum) and CRPs compared to those of the PA. These findings align with previous studies, where CRP levels were used as an objective marker of surgical trauma, reflecting the degree of tissue damage and perioperative stress experienced [15]. Lower CRP levels may indicate reduced tissue injury, which can contribute to less pain, faster rehabilitation, and shorter hospital stays, reinforcing the clinical benefits of the DSA. Moreover, the direct capsular repair used in the DSA contributes to a quicker recovery, a finding that supports the work of Zhang et al., which emphasized the importance of capsule repair for proprioception and joint stability [16].

Furthermore, our results indicated that lower pain levels are associated with a significant reduction in inpatient opioid consumption. Reduced opioid consumption has been associated with improved postoperative recovery across various orthopedic surgeries. In spine surgery, lower opioid use has been linked to reduced complication rates and shorter hospital stays, while in hip and knee arthroplasty, early opioid reduction has been associated with decreased lengths of stay and lower readmission rates [17,18]. Given these benefits, various strategies have been explored to minimize opioid use in the perioperative setting. Among these, spinal anesthesia has been recognized as a safe option in elderly patients undergoing hip surgery, despite concerns about hypotensive events, as demonstrated by Messina et al. in their meta-analysis [19]. While there have been debates about whether the surgical approach used in THA affects opioid consumption, previous studies have been constrained by their inability to adequately control confounding factors [20,21,22,23]. Given the scarcity of studies on opioid consumption in the context of the DSA, we turned to previous studies on the direct anterior approach to identify confounding factors [21]. These included pericapsular injection, opioid naivety, anesthesia, BMI, smoking status, and chronic alcoholism. Our study used PSM to control for significant factors such as age, sex, BMI, chronic alcoholism, smoking status, and underlying diseases when comparing opioid consumption rates. Pericapsular injections were excluded, as all patients received the same morphine injection. After adjusting for confounding factors, differences in early postoperative opioid consumption remained significant. Also, it was observed that the frequency of delirium associated with opioid usage significantly decreased. However, it is noteworthy that this significance diminished 90 days or more after the index surgery. This observation is consistent with other studies, such as that of Gentry et al., which suggests that chronic opioid usage is not associated with the surgical approach used but rather depends on preoperative opioid usage [24].

Our study found no significant differences in overall 90-day readmission rates or other major medical and surgical complications between the two surgical approaches. These results align with those reported by Siljander et al., who found no differences in 30-day major or minor complication rates when comparing different surgical approaches [25]. Additionally, we aimed to assess whether reduced soft tissue damage and direct capsular repair could contribute to a lower dislocation rate. While the dislocation rate in our study was 2.2%, which is lower than that reported with the PA, the difference did not reach statistical significance. This suggests the potential presence of a type II error, indicating that our study may have been underpowered to detect a significant difference. However, clinically, the observed 3.4% difference in dislocation rates may still be meaningful. As this study focused primarily on the impact of the surgical approach, we controlled for variables such as age and head size using PSM. Future large-scale studies with greater statistical power are warranted to further evaluate these findings.

This study had several limitations. First, it was a retrospective comparative study without randomization, potentially making it susceptible to time-dependent and selection biases. Additionally, the average length of stay was longer in both the DSA and PA groups than in other studies. This is primarily because most patients were discharged to their homes rather than to other healthcare facilities. Concerns related to the DSA include the need for special instrumentation and the learning curve associated with mastering the technique. Although we did not directly calculate the learning curve in this study, we believe that the DSA can be easily adopted due to its similarity to the PA, which has been widely adopted in clinical practice. As described in other studies [9,10], the learning curve for the DSA is relatively short, likely due to its technical similarities to the PA. Additionally, the DSA did not require unique instrumentation or offset reamers, unlike the PA [26]. Another limitation of this study is the lack of an evaluation of pre-walking levels and the absence of a precise comparison of the exact time it took for patients to transition from crutches to walking independently after surgery. As a result, it was difficult to make a direct and quantitative comparison of the rehabilitation speed between the two groups, limiting our ability to draw conclusions about the relative speed of their functional recovery. In addition, regarding opioid consumption, while our study found that the DSA resulted in lower opioid use compared to the PA, which aligns with some studies suggesting higher opioid consumption with the PA compared to the DAA, we did not directly compare the DSA with the DAA. Finally, variations in anesthesia choice may have occurred in patients with a history of spine fusion or severe degeneration, as anesthesiologists made decisions based on individual patient conditions.

## 5. Conclusions

The DSA was associated with lower patient-reported pain and lower opioid consumption during hospitalization. This reduction contributed to a lower risk of delirium. In addition, it provided an earlier functional recovery and discharge from the hospital than the PA.

## Figures and Tables

**Table 1:** Baseline demographics.

Characteristic	DSA ^1^, *n* = 244	PA ^2^, *n* = 207	*p*-Value
Age, mean ± SD	60 ± 14	59 ± 14	0.3
Sex, *n* (%)			0.15
Female	131 (54%)	96 (46%)	
Male	113 (46%)	111 (54%)	
Direction, *n* (%)			0.74
Left	113 (46%)	100 (48%)	
Right	131 (54%)	107 (52%)	
BMI, mean ± SD	24.2 ± 3.6	24.6 ± 3.7	0.25
Anesthesia, *n* (%)			0.00
General	139 (57%)	153 (74%)	
Spinal	105 (43%)	54 (26%)	
Diagnosis, *n* (%)			0.4
Avascular necrosis	139 (57%)	134 (65%)	
Osteoarthritis	39 (22%)	39 (19%)	
Secondary osteoarthritis	34 (21%)	34 (16%)	
History of lumbar spine fusion, *n*(%)	20 (8.2%)	10 (4.8%)	0.21
Femoral head size			0.00
28 mm	0 (0%)	2 (1.0%)	
32 mm	50 (20.5%)	48 (23.2%)	
36 mm	122 (50.0%)	147 (71.0%)	
Dual mobility construct	72 (29.5%)	10 (4.8%)	
Opioid naïve patients, *n* (%)	240 (98%)	205 (99%)	0.7
Alcohol abuse, *n* (%)	23 (9.5%)	33 (15.9%)	0.07
ASA ^3^ score			0.00
1	5 (2.0%)	19 (9.2%)	
2	179 (73.4%)	159 (76.8%)	
3	58 (23.8%)	29 (14.0%)	
4	2 (0.8%)	0 (0.0%)	
Underlying disease, *n* (%)			
HTN	100 (41%)	94 (45%)	0.4
Angina	18 (7.4%)	15 (7.2%)	0.99
DM	50 (20.5%)	25 (12.1%)	0.02
Stroke	13 (5.3%)	11 (5.3%)	0.99
Parkinson	3 (1.2%)	2 (1.0%)	0.99
Dementia	4 (1.6%)	5 (2.4%)	0.99
Smoking status, *n* (%)			0.2
Non-smoker	149 (61%)	128 (62%)	
Ex-smoker	39 (16%)	21 (10%)	
Current smoker	56 (23%)	58 (28%)	

^1^ DSA = direct superior approach; ^2^ PA = posterior approach; ^3^ ASA = American Society of Anesthesiologists.

**Table 2:** Baseline demographics after propensity score matching.

Characteristic	DSA ^1^, *n* = 139	PA ^2^, *n* = 139	Abs.SMD ^3^
Age, mean ± SD	58 ± 13	58 ± 14	0.03
Sex, *n* (%)			0.11
Female	67 (48%)	61 (44%)	
Male	72 (52%)	78 (56%)	
Direction, *n* (%)			0.04
Left	64 (45.8%)	67 (48.1%)	
Right	75 (54.2%)	72 (51.9%)	
BMI, mean ± SD	24.5 ± 3.9	24.5 ± 3.3	0.00
Anesthesia, *n* (%)			0.15
General	86 (61.8%)	96 (80.1%)	
Spinal	53 (38.2%)	43 (30.9%)	
Diagnosis, *n* (%)			0.02
Osteonecrosis of femoral head	89 (64.1%)	85 (61.1%)	
Osteoarthritis	26 (18.3%)	32 (23.0%)	
Secondary osteoarthritis	24 (17.6%)	22 (15.9%)	
History of lumbar spine fusion, *n* (%)	7 (5.0%)	6 (4.3%)	0.02
Femoral head size			0.01
28 mm	0 (0.0%)	1 (7.2%)	
32 mm	29 (20.8%)	34 (24.4%)	
36 mm	96 (64.7%)	94 (67.6%)	
Dual mobility construct	14 (10.0%)	10 (7.2%)	
Alcohol abuse	16 (11.5%)	17 (12.2%)	0.03
ASA score			−0.06
1	8 (6.1%)	5 (3.8%)	
2	103 (74.0%)	114 (81.7%)	
3	28 (19.8%)	20 (14.5%)	
Opioid naïve patients, *n* (%)	137 (98%)	137 (98%)	0.000
Underlying disease, *n* (%)			
HTN	54 (38.8%)	56 (40.3%)	0.03
Angina	10 (7.2%)	8 (5.8%)	−0.05
DM	19 (13.7%)	20 (14.4%)	0.02
Stroke	7 (5.0%)	6 (4.3%)	0.00
Parkinson	2 (1.4%)	4 (2.9%)	0.03
Dementia	3 (2.2%)	3 (2.2%)	0.00
Smoking status, *n* (%)			0.10
Non-smoker	93 (67%)	89 (64%)	
Ex-smoker	18 (13%)	13 (9.4%)	
Current smoker	28 (20%)	37 (26%)	

^1^ DSA = direct superior approach; ^2^ PA = posterior approach; ^3^ SMD = standard mean difference.

**Table 3:** Pain-related outcomes with DSA and PA.

Characteristic	DSA ^1^, *n* = 139	PA ^2^, *n* = 139	*p*-Value ^3^
Maximum NRS ^4^			
POD ^5^ #0	5.3 ± 1.4	5.4 ± 1.4	0.684
POD ^5^ #1	4.8 ± 1.1	5.3 ± 1.5	0.006
POD ^5^ #2	4.7 ± 1.1	5.0 ± 1.3	0.112
POD ^5^ #3	4.7 ± 1.1	4.9 ± 1.2	0.20
POD ^5^ #4	4.5 ± 0.8	5.1 ± 1.4	0.000
Minimum NRS ^4^			
POD ^5^ #0	2.9 ± 0.5	3.1 ± 0.7	0.1
POD ^5^ #1	3.0 ± 0.6	3.3 ± 0.9	0.04
POD ^5^ #2	3.0 ± 0.5	3.2 ± 0.8	0.02
POD ^5^ #3	3.0 ± 0.5	3.1 ± 0.7	0.09
POD ^5^ #4	3.0 ± 0.6	3.2 ± 0.7	0.01
OME ^6^, mean ± SD	108 ± 127	194 ± 117	0.002
Opioid refill > 90 days, *n* (%)	2 (1.4%)	1 (0.7%)	>0.9
Opioid refill > 180 days, *n* (%)	1 (0.7%)	0 (0%)	>0.9

^1^ DSA = direct superior approach; ^2^ PA = posterior approach; ^3^ Pearson’s Chi-squared test, independent *t*-test, Fisher’s exact test; ^4^ NRS = Numeric pain rating scale; ^5^ POD = postoperative day; ^6^ OME = oral morphine equivalents.

**Table 4:** Difference in inflammatory-related serum markers between DSA and PA.

Characteristic	DSA ^1^, *n* = 139	PA ^2^, *n* = 139	*p*-Value ^3^
CRP ^4^, Mean ± SD			
POD ^5^ #2	120 ± 50	113 ± 60	0.5
POD ^5^ #5	53 ± 33	74 ± 43	<0.001
POD ^5^ #14	9 ± 16	28 ± 43	<0.001
POD ^5^ #28	7 ± 13	23 ± 39	<0.001
ESR ^6^, Mean ± SD			
POD ^5^ #2	48 ± 29	34 ± 23	0.008
POD ^5^ #5	43 ± 30	42 ± 24	0.6
POD ^5^ #14	41 ± 30	39 ± 24	>0.9
POD ^5^ #28	39 ± 29	38 ± 23	0.8

^1^ DSA = direct superior approach; ^2^ PA = posterior approach; ^3^ independent *t*-test; ^4^ CRP = C-Reactive Protein; ^5^ POD = postoperative day; ^6^ ESR = erythrocyte Sedimentation Rate.

**Table 5:** Difference in postoperative clinical outcomes between DSA and PA.

Characteristic	DSA ^1^, *n* = 139	PA ^2^, *n* = 139	*p*-Value ^1^
Intraoperative Fracture, *n* (%)	1 (0.7%)	1 (0.7%)	>0.9
Revision, *n* (%)	1 (0.7%)	1 (0.7%)	>0.9
Periprosthetic Fracture, *n* (%)	1 (0.7%)	1 (0.7%)	>0.9
Periprosthetic Infection, *n* (%)	0 (0%)	0 (0%)	>0.9
Revision Due to Component Malposition, *n* (%)	0 (0%)	0 (0%)	>0.9
Acute Dislocation, *n* (%)	3 (2.2%)	8 (5.6%)	0.15
Length of Stay, Mean ± SD	5.4 ± 3.1	8.4 ± 8.3	<0.001
HHS ^3^, 6 months post-operation	88.1 ± 11.1	87.3 ± 8.7	0.5
WOMAC ^4^, 6 months post-operation	15.2 ± 11.3	18.9 ± 17.6	0.13
HHS ^3^, 12 months post-operation	93.6. ± 7.9	93.6 ± 3.8	>0.9
WOMAC ^4^, 12 months post-operation	10.2 ± 7.8	9.9 ± 3.8	>0.9
90-Day Readmission, *n* (%)	2 (1.4%)	7 (5%)	0.1
Postoperative Complications, *n* (%)			
Delirium	5 (3.6%)	13 (9.4%)	0.05
Urinary Tract Infection	0 (0%)	0 (0%)	>0.9
Acute Kidney Injury	0 (0%)	0 (0%)	>0.9
Pneumonia	0 (0%)	0 (0%)	>0.9
Stroke	0 (0%)	0 (0%)	>0.9
Sepsis	0 (0%)	0 (0%)	>0.9
Death	0 (0%)	0 (0%)	>0.9

^1^ DSA = direct superior approach; ^2^ PA = posterior approach; ^3^ HHS = Harris Hip Score; ^4^ WOMAC = Western Ontario and McMaster Universities Osteoarthritis score.

## Data Availability

The original contributions presented in this study are included in the article. Further inquiries can be directed to the corresponding author.
